# The mental health of populations directly and indirectly exposed to violent conflict in Indonesia

**DOI:** 10.1186/1752-1505-4-14

**Published:** 2010-07-30

**Authors:** Sherly S Turnip, Ole Klungsøyr, Edvard Hauff

**Affiliations:** 1 Division of Mental Health and Addiction, Institute of Clinical Medicine, Faculty of Medicine, University of Oslo, Oslo, Norway; 2 Faculty of Psychology, Universitas Indonesia Depok, Indonesia; 3 Division of Mental Health and Addiction, Oslo University Hospital Oslo, Norway

## Abstract

**Background:**

Large disasters affect people who live both near and far from the areas in which they occur. The mental health impact is expected to be similar to a ripple effect, where the risk of mental health consequences generally decreases with increasing distance from the disaster center. However, we have not been able to identify studies of the ripple effect of man-made disaster on mental health in low-income countries.

**Objectives:**

The objective was to examine the hypothesis of a ripple effect on the mental health consequences in populations exposed to man-made disasters in a developing country context, through a comparison of two different populations living in different proximities from the center of disaster in Mollucas.

**Methods:**

Cross-sectional longitudinal data were collected from 510 Internally Displaced Persons (IDPs) living in Ambon, who were directly exposed to the violence, and non-IDPs living in remote villages in Mollucas, Indonesia, who had never been directly exposed to violence in Mollucas. Data were collected during home visits and statistical comparisons were conducted by using chi square tests, t-test and logistic regression.

**Results:**

There was significantly more psychological distress "caseness" in IDPs than non-IDPs. The mental health consequences of the violent conflict in Ambon supported the ripple effect hypothesis as displacement status appears to be a strong risk factor for distress, both as a main effect and interaction effect. Significantly higher percentages of IDPs experienced traumatic events than non-IDPs in all six event types reported.

**Conclusions:**

This study indicates that the conflict had an impact on mental health and economic conditions far beyond the area where the actual violent events took place, in a diminishing pattern in line with the hypothesis of a ripple effect.

## Background

A number of factors have been identified as having an impact on the mental health of populations affected by disasters [1-3]. The geographical distance from the centre of the disaster is one of the factors that is likely to influence such an impact. This has been described as the ripple effect of a disaster, and posits that mental health problems spread outward from the center of disaster in a diminishing ripple pattern [4-6]. Disaster spatial zones describe the area at the center of disaster as "area totally destroyed", the immediate area around the disaster center as "partially destroyed area", and the area adjacent to the impact area as the "filter zone" [7]. In populations, exposure level is a fundamental determinant of the mental health effects of disasters [8,9]. Previous studies indicate that those directly exposed to severe incidents are likely to have the highest risk of PTSD and other psychiatric problems [10], and the risk of mental health consequences generally decreases with increasing distance from the disaster agent and decreasing exposure of affected individuals [11].

Man-made disasters often cause more frequent and more persistent psychiatric symptoms and distress than natural disasters [12]. Man-made disasters with a high degree of community destruction, and those in developing countries, are associated with the worst outcomes [13]. A meta-analysis of mental health of displaced persons indicated that internally displaced persons and those who fled due to unresolved conflict were most affected and had the worst mental health outcomes compared to the refugees lived in developed countries [14]. However, most studies on the mental health consequences of disaster have been of natural disasters. They showed that the impact on populations in third world countries differs depending on the proximity to the disaster center [1-3,15]. We have not been able to identify any studies of the impact of man-made disasters on the mental health of indirectly exposed communities in low-income countries. Such information is important in order to assess which populations segments that are in need of different types of assistance.

The present study investigates the impact of long-term violent conflict in Mollucas, Indonesia. The violence, which is believed to be related to religious conflicts between Moslems and Christians, lasted for six years (1999-2005). It spread from its origin in Ambon city to other islands but did not reach a small number of villages in neighboring islands. Although these remote villages were never exposed to direct violence from the Mollucas conflict, reports showed that the non-IDPs living there were affected by it. Indirect effects, such as shortages of life supplies, unavailability of health care, lack of education for children, unverified news related to the conflict and difficulties commuting to other islands and villages due to transportation shortages, were some of the major problems [16]. This paper aims to investigate the hypothesis of a ripple effect on the mental health of populations exposed to violent conflicts by comparing two different populations, namely internally displaced persons (IDPs) who lived in Ambon and were directly exposed to the violence and those who lived in remote villages that had never been directly exposed to violence (non IDPs). We hypothesized that the non-IDPs in remote villages experienced the violent conflict in a pattern in line with the ripple effect, indicated by lower level and lower prevalence of distress, less traumatic experiences and better economic conditions than IDPs living in Ambon. We also hypothesized that there would be different risk factors of distress in both communities.

## Methods

### Study design

This study used cross-sectional data, which were collected as part of a longitudinal study. We compared data from IDPs and non-IDPs, two different types of communities in Ambon, Indonesia, with different proximities to the violent conflict. IDPs data were collected in a longitudinal community based study on Ambon Island over two consecutive years (2005-2006). For this paper, we used data recorded in the second data collection, which was conducted from August to October 2006, and compared it with non-IDPs data collected in September 2006. Ethical clearance was obtained from the Faculty of Psychology, Universitas Indonesia.

### Procedure

Lists of households were requested and obtained from each resettlement and village leader. We randomly selected 471 participants from each list. Details of the randomized sampling procedure used in the study are explained in a previous paper [17]. Local assistants, who were IDPs themselves, underwent specific training for the project and collected the data during home visits. After giving informed consent, the respondents were asked to fill in the questionnaires by themselves, in the presence of an assistant in case the respondent had any questions regarding the items. If a respondent was not capable of completing the questionnaire on his or her own, the assistant would help by reading each item aloud to the respondent and writing down their responses.

### Sample

#### IDPs participant

The inclusion criteria were IDPs living on Ambon Island during and after the violent period who were over 18 years of age and had sufficient competency in Bahasa Indonesia. The exclusion criteria were having a hearing problem, mental retardation or dementia (psychologists' assessment).

Ten locations were selected, based on their accessibility by transportation means from Ambon city, from approximately 85 camps and relocation areas with different living conditions (three temporary camps, two independent relocation areas, three supported relocation areas and two IDPs old land areas) to ensure that all types of IDPs resettlements on Ambon Island were represented. We approached 471 subjects who participated in the first data collection, and 399 subjects agreed to take part in the second data collection in 2006 (83%).

#### Non-IDPs participant

The communities that lived in areas that had never been exposed directly to violent conflict in Mollucas were called non-IDPs. These areas have never been the scene of violent conflicts and therefore do not have any IDPs, probably due to the homogeneity of religion among the inhabitants. A cross-sectional data collection was carried out in Buru and Saparua Islands in the archipelago of Mollucas province in September 2006. Both islands are located approximately 300-400 kilometers from Ambon, and it took up to 15 hours to reach them with ferries and cars. One village was chosen from each island; Booi village on Saparua Island, and Kayeli village on Buru Island. Those two villages were selected because there had not been had any incident related to the Mollucas conflict within the village, less than 5% of their inhabitants had participated actively in the Mollucas conflict outside their own village (according to information from local district and village leaders), and they were geographically accessible by public transportation. Inclusion criteria for participants were that they had lived in the selected villages during and after the violent period (in the past six years), were over 18 years of age, had never been actively involved in the Mollucas violent conflicts, were sufficiently competent in Bahasa Indonesia, and did not have hearing problems, mental retardation or dementia (psychologists' assessment). We collected the names of the villagers from the village leaders and randomly picked 120 participants in both villages. Of the 120 people we approached, 111 agreed to participate in the study (93%).

### Measures

#### Demographic section

**Figure 1:**
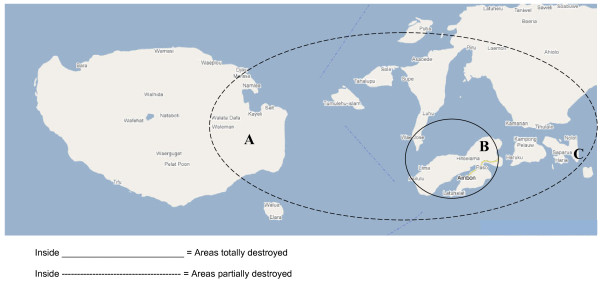
**Violent conflict spatial zones in Ambon, Buru and Saparua Islands**. A: Kayeli village in Buru Island. B: Ambon city in Ambon Island. C: Booi village in Saparua Island

This section measured basic demographic information including age, gender, education, displacement status, marital status, religion and address. The map of the study area is presented in Figure 1.

#### Psychological distress

The Hopkins Symptoms Check List-25 (HSCL-25) was used to measure psychological distress in the past week [18]. Items are rated on a scale ranging from 1 (not at all) to 4 (extremely). This instrument has been widely used in studies of forced migrants in different countries [19], including IDPs in low-income countries [19]. We used the conventional criteria for determining "caseness" on the HSCL-25 measure; a score ≥ 1.75 was taken as an indication that the person probably needed a further diagnosis of psychological distress [20-22]. The details of the cultural validation of the HSCL-25 in the Indonesian setting have been described in another paper [17].

#### Sense of Coherence

The Sense of Coherence (SOC) is used as an indicator of resiliency. The SOC is a generalized, long-lasting view one has of the world and of living in the world. This concept has three aspects: comprehensibility, manageability and meaningfulness [23,24]. Some comparable concepts associated with resiliency were "hardiness" from Kobasa, "sense of permanence" from Boyce Thomas, domains of social climate from Rudolf Moos, and family's construction of reality from David Reiss [23].

We used the short version of the Sense of Coherence questionnaire (SOC), which consists of 13 items. The SOC scale is a seven-point semantic differential Likert scale. Conventionally, each item is scored from 1 to 7 for "positively" formulated items, with "negatively" formulated items scored reversely. The scores are then added to get the SOC score; a higher score indicates higher SOC [23]. The SOC 13 has been used in low-income countries and in postwar settings [24,25]. Before collecting the data, we conducted a cultural validation using the translation monitoring form [26]. The process includes the translation and back translation by different persons followed by comparison of the two translation results by a bilingual mental health professional, evaluation of the local language translation by focus group discussion of lay people and pilot study. In this study, a five-point scale was chosen because it was strongly advised by participants of the focus group discussion (FGD) during the cultural validation. The sum of all items was then multiplied by 7/5 to make the total score comparable to other results [24]. This cultural validation process ensured the relevance and meaningfulness of the sense of coherence concept in the local culture in Mollucas. Preliminary interviews with traditional leaders showed that Mollucans believed in their ability to rebound from difficulties. A Mollucan was described to have similarities with the sago tree: rough outside but white inside. This symbol represents the characteristics of Mollucans as being tough and resilient with purity and sincerity at heart [27].

#### Traumatic experiences

Participants were asked about their traumatic experiences during the conflict period. The questions were derived from the most common traumatic experiences among IDPs in Ambon: witnessing murder, feeling that one's life was ever in danger, witnessing violence toward people and/or property, having a close family member who died due to the conflict and being injured herself/himself due to the conflict. Those experiences were identified through several focus group discussions (FGD) and interviews with IDPs in Mollucas. All of the questions were formulated as 'yes' or 'no' questions.

#### Economic conditions

We developed our socioeconomic and demographic questionnaire in Ambon, based on the indicators from the National Socio Economic Survey in Indonesia [28]. The poverty level was measured by three variables. The first was a structural variable that consisted of five items comprising educational level, disruption at school during the conflict period, employment status and income and gifts received from outside the household in the past three months. The second was a consumption variable that consisted of five food items and four nonfood items designed to differentiate between the well-off and the poor. The last was an asset ownership variable that consisted of the 10 items that best defined one's socioeconomic status in the local setting. The details of development of this instrument are given in a previous paper [17].

### Statistical analysis

We conducted chi square tests to identify differences in demographic characteristics and traumatic experiences with respect to displacement status. In order to identify differences in distress scores and economic condition indicators, we conducted independent group *t*-tests between IDPs and non-IDPs. Since we had the IDPs data from two consecutive years, we also conducted paired group t-tests within the IDP group on those two occasions. Proportions of distress "caseness" were compared between IDP and non-IDPs through logistic regression analysis with psychological distress (case vs. noncase) as the dependent variable, and the status (IDPs vs. non IDPs) as independent variable.

The focus was the association between displacement status and psychological distress. Various background factors were considered to be potential confounders and adjusted for. We entered the background factors as independent variables one by one into bivariate regression analysis and retained all variables that were significant at p ≤ 0.1 for the multiple regression analyses. Then all possible interactions and non-linearities were assessed and also retained for multiple regression analysis at p ≤ 0.1. Model selection was done by comparing different combinations of covariates in a stepwise fashion and choosing the best-fit model. Only significant terms were kept in the final model. We used SPSS version 14 for statistical analysis. All significance tests were two-sided with significance levels of 0.05 [29].

## Results

### Demographic characteristics and traumatic experiences report

The numbers of female and male participants in both IDPs and non-IDPs groups were almost equal. The participants in the IDPs group were 19-81 years, with a mean age of 39 years (SD = 14.2), and participants in the non-IDPs group were 18-79 years, with a mean age of 43 years (SD = 16.2). There was a significantly higher percentage of Christian participants in the non-IDP group than in the IDPs, and a significantly larger percentage of IDPs participants had a higher level of education than non-IDPs participants. There was significantly higher percentage of married people among the non-IDPs compared to the IDPs. Other demographic characteristics of the participants are presented in Table 1.

**Table 1:** Demographic characteristics of communities affected directly and indirectly by violent conflict in Mollucas

	IDPs (%) *N *= 399	Non-IDPs (%) *N *= 111	χ^2^	*p*
Gender				
Female	235 (59)	59 (53)	1.174	0.279
Male	164 (41)	52 (47)		
Age				
< 30 years	118 (30)	27 (24)	1.176	0.278
≥ 30 years	281 (70)	84 (76)		
Religion				
Christian	224 (56)	78 (70)	7.179	0.007
Moslem	175 (44)	33 (30)		
Education				
< 9 years	144 (36)	60 (54)	11.179	0.001
≥ 9 years	255 (64)	51 (46)		
Marital status				
Married	304 (76)	94 (85)	129.40	< 0.001
Not married	95 (24)	17 (15)		

The comparison of the number of IDPs and non-IDPs participants who experienced traumatic events is presented in Table 2. Significantly higher percentages of IDPs experienced each of the six kinds of traumatic events reported than non-IDPs. The largest difference was that more than half of the IDPs group reported having witnessed violence toward property while only 4% of the non-IDPs group had. The traumatic event most commonly reported by IDPs and non-IDPs was feeling threatened. The event least commonly reported by IDPs was being injured in the conflict, while in the non-IDPs group the least experienced event was witnessing murder.

**Table 2:** Comparison of traumatic experiences between communities affected directly and indirectly by violent conflict in Mollucas

	IDPs (%) *N *= 399	Non-IDPs (%) *N *= 111	χ^2^	*p*
Threat	361 (77)	46 (41)	52.944	< 0.001
Injured	42 (9)	3 (3)	4.863	0.027
Witnessed murder	135 (29)	1 (1)	38.662	< 0.001
Witnessed violence toward people	181 (38)	5 (5)	47.543	< 0.001
Witnessed violence toward property	238 (51)	4 (4)	81.437	< 0.001
Family death	208 (44)	19 (17)	27.616	< 0.001

### Mental health and economic conditions

The mean score of psychological distress in 2006 for the total sample was 1.68 (SD = 0.46). The comparison of mental health indicators and economic conditions between IDPs and non-IDPs is presented in Table 3. There was no significant difference in crude distress level between IDPs and non-IDPs. We had data for the IDPs distress score one year previously, which was 1.78 (SD = 0.50), significantly different from the distress level of both IDPs and non-IDPs in 2006 (*p *= 0.001 and < 0.001 respectively). In the non-IDPs group there was no significant gender difference in psychological distress (p = 0.085). The distress mean scores for females were 1.66 (*SD *= 0.47) and for males were 1.56 ([*SD *= 0.43). In the IDPs group, females had significantly higher distress levels than males (p < 0.001), where the distress mean scores were 1.78 [SD = 0.47] and 1.58 [SD = 0.44] respectively.

**Table 3:** Comparison of mental health indicators and economic conditions between communities affected directly and indirectly by violent conflict in Mollucas

	IDPs mean scores (*SD*)	Non-IDPs mean scores (*SD*)	Range of scores	*p*
Psychological distress	1.70 (0.47)	1.62 (0.46)	1-4	0.112
Sense of coherence	65.3 (9.5)	65.7 (10)	18-91	0.682
Structural variable	13.3 (4.6)	14.7 (2.3)	2-24	< 0.001
Consumption variable	10.3 (4.5)	10 (3.9)	0-22	0.370
Asset ownership variable	12.2 (6.3)	15.5 (7.1)	0-31	< 0.001

There was significantly more "caseness" of psychological distress in IDPs than in non-IDP (OR = 1.6, *p *= 0.042) as presented in Table 4. There was no significant difference in sense of coherence between IDPs and non-IDPs.

**Table 4:** Prevalence and odds ratio of distress cases among communities affected directly and indirectly by violent conflict in Mollucas

	IDPs (*N *= 399)	Non-IDPs (*N *= 111)	*p*
Distress "caseness"			
Frequency	165	34	0.041
Percentage	41	30	
Odds ratio	1.6	1	0.042
95% CI	1.1-2.5		

Notes

CI: Confidence interval

Economic conditions of non-IDPs were significantly better than IDPs with regard to the structural and asset ownership variables (*p *< 0.001 for both variables), and there was no significant difference in consumption between IDPs and non-IDPs.

### Risk and protective factors of distress

**Figure 2:**
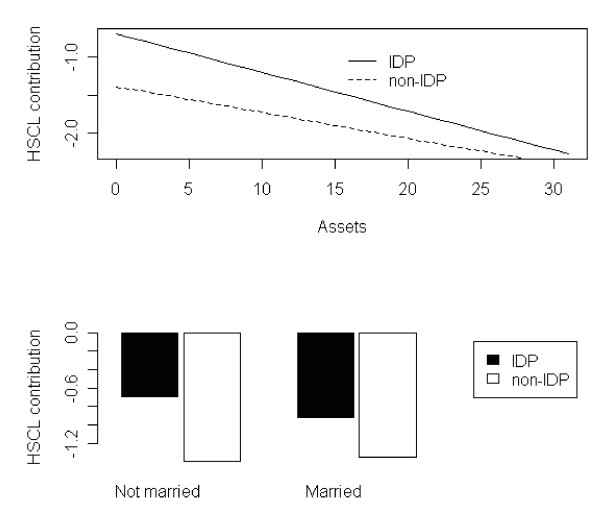
**Effect modification of displacement-distress relation by assets (upper panel) and marital status (lower panel)**.

The regression model explained 23.6% variance of psychological distress (Table 5). The variable with the largest contribution to explained variance was SOC (6.7%), followed by gender (3.9%) and status of being IDPs or non-IDPs (2.8%). Being IDPs was a risk factor for distress, while higher SOC was a protective factor. Other risk factors for distress were being female, not being married (single and widowed), owning fewer assets and feeling that one's life was in danger. Interaction between lower SOC and lower number of assets was a significant risk factor for distress. Significant interactions were found between SOC and asset ownership, asset ownership and displacement status, and marital status and displacement status. Owning fewer assets and not being married showed a stronger negative association with the distress levels of IDPs than non-IDP. IDP with fewer assets were more distressed than non-IDPs with fewer assets (Figure 2, upper panel) and IDP who were not married were at an higher risk of distress than their married counterparts and non-IDPs (Figure 2, lower panel)

**Table 5:** Multiple regression analysis of risk factors of psychological distress for communities affected directly and indirectly by violent conflict in Mollucas

Variables	Total sample (*N *= 510)		
	
	B	95% CI	*p*
Intercept	4.607	3.907-5.305	< 0.001
Displacement status (IDPs)	-.695	-1.060-(-0.331)	< 0.001
Gender (male)	-.167	-0.241-(-0.094)	< 0.001
Threat	.105	0.021-0.188	0.014
Assets (higher)	-.068	-0.111-0.025	0.002
SOC (higher)	-.024	-0.033-(-0.016)	< 0.001
Married	-.479	-0.768-(-0.189)	0.001
Assets * SOC	.001	< 0.001-0.001	0.021
Assets * Displacement status	.017	0.004-0.029	0.010
Marital status * Displacement status	.260	0.026-0.495	0.030

## Discussion

### The ripple effect of violent conflict

Our study found that the prevalence of psychological distress in a population indirectly affected by violent conflict was significantly lower than in a population in the same region that was directly affected. This confirmed our hypothesis that there would be a ripple effect of disaster across different proximities to violent conflict; our findings revealed that people who lived in exposed areas had higher distress levels, which was in line with the results of other studies [4-6,30]. Figure 1. presents the map of areas affected by the violent conflict in Mollucas province. The results supported our hypothesis that non-IDPs experienced less traumatic events than IDPs. However, because four in ten participants felt that their lives were threatened, and one in eight had lost a close family member, the consequences of the violence for the non-IDPs was still considerable. These events can be experienced regardless of one's physical proximity to the conflict. Non-IDPs were still exposed to the conflict by news delivered to them by television and radio stations, newspapers, relatives living in Ambon and people who had traveled to the area of conflict. Exposure through various media can lead to the perception of frightening and life-threatening events when an individual personalizes the events or views themselves as a potential victim [31,32].

The hypothesis of a ripple effect on the economic conditions was partially confirmed. The non-IDPs had significantly higher levels of structural resources and asset ownership than the IDPs. The non-IDPs lived in conflict-free areas, so their houses, land and other assets stayed intact. On the other hand, most of the IDPs had lost their assets, such as houses and land, during the forced migration that followed the conflict [16]. IDPs also experienced more disturbance of education than non-IDPs, which may lead to school dropouts and job loss. However, both population groups had similar levels of food and nonfood consumption. Most people in Mollucas are still fulfilling their daily consumption needs from their environment. Many people grow their own vegetables or catch fish for their own families [33,34]. Furthermore, it was equally difficult for IDPs and non-IDPs to obtain nonfood consumption objects such as clothes, health care and recreation equipment. The Mollucas are located far from the big cities, and goods transportation could be problematic during the conflict and post-conflict period due to the destruction of economic infrastructures [35].

The insignificant difference of distress level between IDPs and non-IDPs in 2006 was probably related to the improvement of the economic conditions in the IDPs areas (which were in the main island of Ambon) after the peace condition resumed in 2005. Being the location of the offices of the provincial government, Ambon island had received substantial economic stimulation activities which generated better income for most of its inhabitants. The security level had also improved as to attract national and foreign investors to the island. Numerous IDPs resettlement project have been conducted to speed up the recovery process. Those improved conditions might be associated with the significant reduction of the IDPs distress level from the previous year, and became more similar to those of the non-IDPs. On the other hand very little had changed in the two islands where the non-IDPs respondents lived after the conflict had ended. Progress in development was very slow and people live the same kind of life that they have endured since previous year, possibly even years before.

Although there were significant differences between IDPs and non-IDPs proportions in religions and educational level, we found no associations between these variables and mental health in either community. Therefore those demographic characteristics are not likely to be associated with the psychological distress.

### Risk factors for psychological distress

IDPs status appears to be an important risk factor for distress, both as a main effect and an interaction effect. This result indicated that there was a ripple effect from the disaster, where closer proximity to the violent conflict would predict higher distress. The main difference between IDPs and non-IDPs living conditions was the proximity to the violent conflict and their exposure to it. Previous studies demonstrated significant effects of proximity to the epicenter of disaster on morbidity rates and degree of psychological distress [1,36]. A study on nationwide psychological responses to the September 11 terrorist attack in New York indicated that people directly exposed to the event had significantly higher post-traumatic stress symptoms than those indirectly exposed [30]. As we found in our sample, those who are indirectly exposed can be expected to show a lower prevalence of psychiatric problems [37].

Being female was a risk factor for distress for the combined sample. Previous epidemiological studies in general populations have shown that women suffer more anxiety and depression than men [38]. In a post-conflict situation like Mollucas, women often experience more upsetting life events and are more vulnerable to abuse, which may be related to adverse life conditions, especially during the violence period [39-41]. Qualitative inquiries indicated that although women did not suffer of any gender based violence in the Mollucas conflict, many of them have been the victims of increased domestic violence since the conflict had started. In times of hardship, women often have more responsibilities and burdens in domestic areas, such as expanding their social role and entering the job market. The role-related overload of responsibilities that women have to endure might contribute to the elevated risk of common psychological distress [40,41].

Feeling that one's life was threatened was the traumatic event most commonly reported by IDPs and non-IDPs, and a risk factor for distress for both groups. Exposure to the disaster, regardless of the distance from it, awoke more intense fears of being the victim of violence and created distress among people [31,32]. We found that the prevalence of the fear of losing one's life was lower in the non-IDP than in the IDPs (41% and 77% respectively), but it was a risk factor for distress in the combined sample.

A lower score on asset ownership was a risk factor for distress. However, results from the analysis of interaction effects indicated that fewer assets among IDPs were associated with higher distress than among non-IDPs. Most of the IDPs had lost their assets during the violent conflict, and this made life more difficult. IDPs with the fewest assets lived in greater poverty than other IDPs. Fewer assets could mean that IDPs did not have a place to live or land to cultivate.

Another significant risk factor for distress was marital status. Those who were not married had higher distress levels than those who were married. From the interaction effect between marital status and IDPs status, we found that IDPs who were not married were at an even higher risk of distress than their married counterparts and non-IDPs. Previous studies have found that being single or having been formerly married were risk factors for depression and anxiety symptoms [42,43].

Sense of coherence appeared to be a protective factor from psychological distress for the combined sample of IDPs and non-IDPs, both as main effect and as an interaction effect with increasing number of assets. One might expect a higher SOC in a more stabile population (non-IDPs), which we did not find. SOC is stabilized around the age of 30 and it is considered as a trait [23]. The mean ages were 39 and 43 years for IDPs and non-IDPs respectively, and therefore SOC is likely to be a stable trait for them. Although both communities felt threatened by the conflict, their SOC appears not to be affected by it. This finding complements previous studies that revealed a strong association between SOC and positive outcome of mental health [44]. People with a high SOC can cope with stressful situations better and stay healthier than those with a low SOC [44,45]. The interaction between asset ownership and SOC indicated that people with lower SOC and fewer assets had the highest distress in our sample.

### Limitations

We obtained information about traumatic events at different times for the two sample groups. The first time we collected data from the IDPs was in 2005, one year after the last major violent incidents took place in Ambon. We collected data from non-IDPs in 2006, when we conducted the second data collection from the IDPs. The time interval between the last violent conflict and data collection may have caused some memory distortion regarding the questions of traumatic experiences among non-IDPs. The distortion might have caused underreporting of traumatic events, and may make comparison with IDPs more difficult. The difference in psychological distress we found might have been more pronounced if we had obtained data from non-IDPs in 2005.

Another limitation is that we only collected data from two communities in the province of Mollucas. Therefore, we cannot analyze the ripple effect on people living further from Ambon, such as those who live in the more distant neighboring provinces.

### Strengths

The main strength of this study is the uniqueness of the data. As far as we are aware, it is the only study of the ripple effect on mental health from a man-made disaster with a high degree of community destruction in a developing country, despite the suggestion that such disasters in low-income countries are associated with the worst outcomes of mental health [13].

The instruments used in this study to measure mental health and socioeconomic conditions had been culturally validated. The details of the development and validation of the instruments have been described in another paper [17]. Benefits resulting from this cultural validation were probably the low refusal rate (17% and 7% among IDPs and non-IDPs respectively) and positive feedback from respondents. The cultural validation is a key issue in enabling this study to obtain meaningful and high quality results.

The different response rates between the IDP and non-IDP samples (83% and 93% respectively) were due to conditions in the field. The IDPs sample list was based on the list from the previous year and therefore was not based on an updated list of the inhabitants in the study areas. The research team could not find 57 participants due to their movement to areas outside Mollucas or unknown address in Mollucas, and 4 participants had passed away during the time interval between 2005 and 2006. Only 11 participants refused to take part with various reasons (severe illness, very busy with their activities, could not find a convenient time to participate). On the other hand, we had an updated list of villagers for the non-IDPs sample comprised of the actual people lived in the village during the data collection. Therefore the return rate was much higher in the non-IDPs sample. However, there is no assumption of information bias since we did not find any significant difference of distress between the IDPs who participated in the second year study and those who were not.

### Implications

This study has general implications for emerging theories of the impact of violence and disasters on communities. The impact of threat may be expanded to a wider range of communities in varying degrees of proximity from the places where the conflict originated. The individual level measurements used in this study were essential since the appraisal of the impact of a violent conflict depends on individual perception although the exposure was a collective and ecological one. Individual responses toward one exposure may vary depending on many factors such as previous life experiences, the meaning given to the exposure by the person, individual losses due to the exposure, etc. Our findings are in line with the systemic or ecological contextual approach that indicates that the impact of violence toward an individual or a community is transposed to the society at large (macro-level) [2]. The different levels of society are interwoven and linked to each other so that violence rarely occupies the directly affected area only [2]. When one approaches disaster areas, one may use the findings of our study to provide assistance to the different levels of society.

This study can serve as a basis for more research. Extension of the study, such as the expansion of the sampling areas to other parts of Indonesia, might improve the generalizability of the hypothesis of a ripple effect of a disaster on psychological distress.

## Competing interests

The authors declare that they have no competing interests.

## Authors' contributions

SST: Did data collection, data analysis and drafted the manuscript. OK: Involved in data analysis and in writing the manuscript. EH: Involved in data collection, data analysis and writing the manuscript. All authors read and approved the final version of the manuscript.
